# General Pathology

**Published:** 1886-11

**Authors:** 


AN INTRODUCTION TO GENERAL PATHOLOGY. *Founded on Three Lectures delivered at the Royal College of Surgeons, London, 1886, by* JOHN BLAND SUTTON, F.R.C.S., ETC. *Philadelphia*: P. BLAKISTON, SON & CO., *pp. 390.*

The author says, "the object of these lectures is to endeavor to deal with the enormous collection of facts, gathered together under the term Pathology, by the application of the fundamental principles of the doctrine of evolution."

The work might have been called, with propriety, an Introduction to Comparative Pathology, since the author strives to throw light upon the most general pathological processes by examples drawn from all classes of animals. He has been particularly well prepared for this work by his education and constant study of comparative anatomy and pathology. The author has written a book that will be novel as well as exceedingly interesting to most practitioners. Very much light can be thrown upon pathological processes, that in man are dark and obscure, by the study of the same or homologous processes in lower animals, and it is this that our author has striven to accomplish.

The chapters that are particularly interesting are those on inflammation and tumors. In connection with inflammation the author explains the true rôle of the white blood-corpuscle in promoting absorption of solids by intracellular digestion, as do no other authors of text-books. In regard to the etiology of tumors, he adopts fully Conheim's view that minute islands of embryonic tissue exist and form the nuclei of neoplastic growths. Numerous illustrations are cited that seemingly substantiate this theory.

The last chapter discusses "Pathology and the part it has played in evolution."

The entire book is interesting. The illustrations are new and novel and well chosen. The work therefore deserves to be widely read. N. S. D., JR.

